# A 16‐Year‐Old Boy With ARDS and Septic Shock, an Atypical Presentation of Murine Typhus: Case Report and Review of the Literature

**DOI:** 10.1155/crcc/1267884

**Published:** 2026-09-10

**Authors:** Pierre H. François, Thalia Leclerc, Manon Jaboyedoff, Onya Opota, Gilbert Greub, Guillaume Maitre, Vivianne Chanez

**Affiliations:** ^1^ Pediatric Intensive Care Unit, Department Women–Mother–Child, Lausanne University Hospital, Lausanne, Switzerland, chuv.ch; ^2^ Unit of Pediatric Infectious Diseases and Vaccinology, Service of Pediatrics, Department Women–Mother–Child, Lausanne University Hospital and University of Lausanne, Lausanne, Switzerland, chuv.ch; ^3^ Institute of Microbiology, Lausanne University Hospital and University of Lausanne, Lausanne, Switzerland, chuv.ch; ^4^ Infectious Diseases Service, Department of Internal Medicine, Lausanne University of Lausanne, Lausanne, Switzerland

**Keywords:** ARDS, case report, child, murine typhus, septic shock

## Abstract

Murine typhus is a flea‐borne disease caused by the bacteria *Rickettsia typhi* that occurs worldwide, but predominantly in endemic areas. The clinical presentation of typhus is often nonspecific, leading to delays in diagnosis and treatment. The severity of the disease varies widely. Most cases are relatively mild, but some patients develop a very severe form and may die. We report a case of severe murine typhus complicated by septic shock, alveolar hemorrhage, and acute respiratory distress syndrome in a teenager who was born in Somalia and had immigrated from Kenya to Switzerland. Diagnosis was suspected upon admission in the pediatric intensive care unit and was confirmed by PCR. The patient improved with doxycycline treatment. Rickettsial disease should be considered in the differential diagnosis of patients returning from endemic areas, and anti‐rickettsial treatment should be started without delay to ensure rapid recovery and avoid severe complications.

## 1. Introduction

Murine typhus is a febrile disease caused by *Rickettsia typhi*, an obligate intracellular Gram‐negative bacterium. It is primarily transmitted from rodents to humans through fleas, although other reservoirs and vectors are also reported [1]. *R. typhi* is present worldwide, particularly in warm climates such as North and South America, Southeast Asia, Africa, Australia, and southern European countries. In Europe, it is found in Mediterranean countries, with important series of endemic cases in Spain, Croatia, Greece, and Cyprus, and only a few imported cases observed in most other European countries [2]. In Switzerland, all cases are imported [3]. Murine typhus cases are also occasionally observed among international travelers returning from abroad [4].

Murine typhus, which generally presents as an acute undifferentiated febrile illness, poses a significant diagnostic challenge. As it presents with a range of nonspecific symptoms, including fever, headache, cough, myalgia, arthralgia, rash, diarrhea, and nausea, the disease is often misdiagnosed (especially in the absence of rash), resulting in an underestimation of its incidence [1]. Murine typhus is typically a mild disease, although some patients develop septic shock and multiorgan failure.

Here, we report a case of severe murine typhus in an adolescent who had recently immigrated from Kenya to Switzerland, complicated by septic shock and acute respiratory failure.

## 2. Case Report

A 16‐year‐old adolescent was admitted to the pediatric intensive care unit (PICU) for respiratory failure and septic shock after a 7‐day history of fever, chills, headaches, abdominal pain, vomiting, cough, and hemoptysis. He was born in Somalia, lived in Kenya for many months, and arrived in Switzerland 2 weeks before the onset of symptoms. He described his migration journey from Kenya as long but without major difficulties. However, he mentioned staying in a refugee camp where access to sanitation facilities was limited, and living conditions were precarious. He was otherwise in good health, and his vaccinations were up to date to the age of 1 year old. Four days before PICU admission, he visited an adult emergency department with nuchalgia and severe occipital headaches radiating temporally for 3 days; these were stable in intensity and partially relieved by paracetamol. He also presented with visual troubles, dizziness, chills, fever, and anorexia. There was no history of bites or animal exposure, and the clinical examination was normal. Laboratory tests (Table 1) showed a mild inflammatory syndrome, mild leukopenia, and mild thrombopenia. He left the emergency room with a diagnosis of viral infection and symptomatic treatment (paracetamol and ibuprofen). The next day, he developed basithoracic and abdominal pain (right hypochondrium and epigastrium) with repeated food vomiting and two watery stools with mucus but without blood. Two days before his admission, his general condition worsened with cough, hot–cold sensation, and night chills. The day before admission, he developed dyspnea; upon admission, he also presented with hemoptysis.

**Table 1:** Evolution of *Rickettsia* spp. and typhus group *Rickettsia* real‐time PCR in the different samples and evolution of the serology titers, as well as different biological parameters and all the microbial tests performed.

		Day −4^a^	Day 0^b^	Day 1	Day 2	Day 3	Day 4	Day 5	Day 6	Day 7	Day 8	Day 9	Day 15
*Rickettsia* spp. PCR (i) (copies/mL)	Blood		29,469		5380	2769	2717	2765					
	Endotracheal aspiration			5781									
	Pleural effusion			57,590	7471	4229	1090	608					

Typhus group PCR (ii) (copies/mL)	Blood		21339		3329	3237	3308	2758	556	284	127	0	
	Endotracheal aspiration			3217									
	Pleural effusion			28,415	4781	5014	2378	448	156				

Serology titers	*Rickettsia typhi* IgM			1/200								1:6400	1:1600
	*Rickettsia typhi* IgG			1/80								1:10,240	1:10,240
	*Dengue* (*IgM and IgG*)			Negative									

Cultures	Blood (aerobic and anaerobic)		Sterile	Sterile				Sterile					
	Endotracheal aspirations				Sterile								
	Pleural effusion				Sterile								
	Urine							Sterile					

Laboratory tests (units) [normal values]	Platelet (G/L) [150–350]	125	29	25	22	35	55	75	133		298		
	Leucocyte (G/L) [4–10]	3.6	4.6	8.6	11.1	8.4	6.9	7.0	9.2		9.4		
	Lymphocyte (G/L) [1.5–4.0]		0.71	0.95	1.11	0.67	1.24	0.91	1.56		3.1		
	C reactive Protein (mg/L) [< 5]	60	304	229	291	167	74	40	47	29	16		
	Creatinine (*μ*mol/L) [50–100]	75	102	65	53	50	40	37	39	40	49		
	Urea (mmol/L) [2.6–6.8]	3.8	11.3	5.6	4.6	6.1	6.9	5.5	5.5	4.8	5.8		
	ASAT (U/L) [5–35]	37	541	228	171	128	129	81	75	83			
	ALAT (U/L) [10–40]	20	347	178	126	101	108	99	104	106			
	Total bilirubin (*μ*mol/L) [0–21]	5	30	17	19	9			20	12			
	Direct bilirubin (*μ*mol/L) [0–10]		22	15	17	8			7	5			
	GGT (U/L) [10–70]	36	130	110	86	119	162	163	210	188			
	Fibrinogen (g/L) [1.9–4.1]		4.98	3.7	3.5	2.1							
	INR		1.1	1.1	1	1							
	aPTT (s) [24–38]		56	54	49	35							
	D‐dimer (*μ*g/L) [< 500]		35,200										
	Na (mmol/L) [135–145]	136	140	142	143	147	149	148	141	136	134		

*Note:* (i) Pan‐*Rickettsia* PCR targeting both the typhus group Rickettsia and the spotted fever group rickettsia. (ii) Typhus group PCR amplify *Rickettsia typhi* and *Rickettsia prowazekii* DNA.

^a^First emergency room visit.

^b^At the emergency room or at the admission in the PICU.

In the adult emergency room (Day 0), he presented with hypotensive shock that normalized after administration of 3 L of Ringer lactate. He also presented with hypoxemic respiratory distress. The abdomen was bloated and painful in the epigastric and right hypochondrium with defense and a positive Murphy′s sign. He exhibited hepatosplenomegaly.

Laboratory findings revealed lactic acidosis (venous pH 7.3, CO_2_ 49 mmHg [N 40–50], bicarbonate 21 mmol/L [N 22–26], lactate 4.9 mmol/L [N < 2]), severe thrombocytopenia (29 G/L), lymphopenia (0.71G/L) with a normal leukocyte count, a severe inflammatory syndrome (C reactive protein 304 mg/L [N < 10]), acute renal failure (creatinine 10.2 *μ*mol/L [N 50–100], urea 11.3 mmol/L [N 2.6–6.8]), normal natremia (Na 140 mmol/L [N 135–145]), and hepatic cytolysis (10x normal value) with cholestasis. Clotting test showed hyperfibrinogenemia (4.98 g/L), high activated partial thromboplastin time (APTT of 56 s), and high D‐dimer values (35,200 *μ*g/L) (Table 1). Blood and urine cultures were taken, and then, the patient received antibiotic therapy with meropenem, clarithromycin (for a possible agent of atypical bacteria such as *Legionella*), and artesunate.

**Figure 1:**
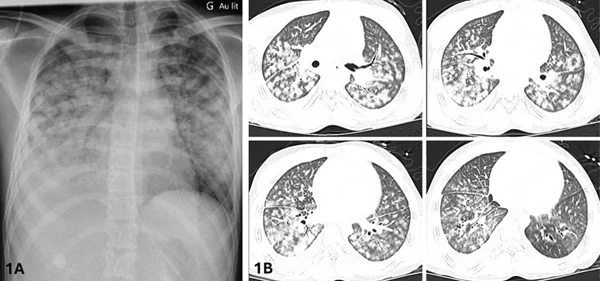
(A) Chest x‐ray on PICU admission showed diffuse bilateral air‐space opacities with bronchogram and right pleural effusion. (B) Thoracic CT scan showed bilateral and symmetrical diffuse alveolar opacities suggestive of alveolar hemorrhage and areas of ground glass suggestive of underlying infection and pleural effusions.

He was transferred to the PICU for septic shock of unknown origin. Upon arrival, he presented with severe acute respiratory failure with hemoptysis. Because of lack of improvement on noninvasive ventilation, he was intubated a few hours after his arrival. He quickly developed severe acute respiratory distress syndrome (ARDS). Chest x‐ray showed an alveolar infiltrate (Figure 1A). A thoracoabdominal CT scan (Figure 1B) showed bilateral and symmetrical diffuse alveolar opacities suggestive of alveolar hemorrhage and areas of ground glass suggestive of an underlying infection, as well as pleural effusions, hepatomegaly, and perivesicular oedema. Protective ventilation was introduced with prone positioning sessions.

At the same time, he needed aminergic support with high doses of norepinephrine and hydrocortisone treatment. Echocardiography did not reveal any cardiac dysfunction. He presented a macular rash (gooseflesh appearance with small, dark, punctiform lesions, slightly raised, without erythema, petechiae, or purpura) on his trunk, palms, and soles. Antibiotic therapy was extended upon admission with vancomycin (to cover a possible MRSA) and doxycycline (to cover possible rickettsiosis or leptospirosis, given the compatible history and clinical presentation). Since an agent of atypical pneumonia was less likely and since it would have also been covered with doxycycline, the clarithromycin treatment was stopped. Artesunate was stopped after negative malaria test results (negative immunochromatography, microscopy, and polymerase chain reaction [PCR]). The large right pleural effusion was drained 1 day after admission, revealing a serobloody exudate.

**Figure 2:**
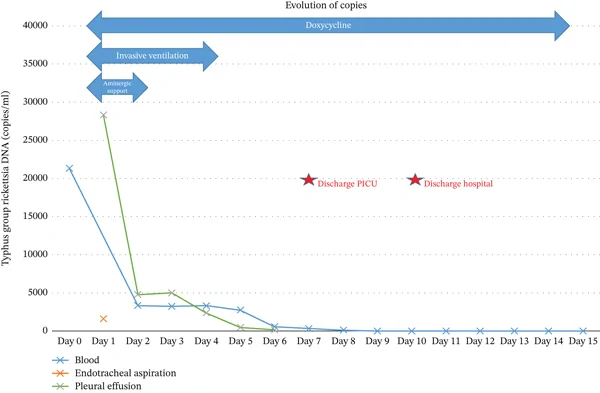
Evolution of the typhus group *Rickettsia* PCR in the different samples.

We performed a large microbiological test (Table 2) workup on blood, endotracheal secretions, and pleural fluid with cultures, several serological assays and several bacterial PCR including a duplex real‐time PCR assay targeting *Rickettsia*: (i) a genus‐level pan‐*Rickettsia* targeting both the typhus group Rickettsia and the spotted fever group rickettsia, hereafter called *Rickettsia* spp. PCR and (ii) a typhus group PCR that may amplify *R. typhi* and *Rickettsia prowazekii* DNA, if present in a clinical sample. By Day 2, this investigation revealed a very high burden of typhus‐group *Rickettsia* DNA in all tested specimens (Table 1). Serology taken the day after admission (Day 1) was weakly positive for *R. typhi* (IgM 1/200 [N < 1/200], IgG 1/80 [N < 1/40]) and that taken a week later (Day 9) strongly positive (IgM 1/6400, IgG 1/10240) (Table 1). As all microbiological cultures were sterile, meropenem, and vancomycin were stopped. The evolution was favorable, allowing cessation of aminergic support on Day 3 and extubation on Day 5, with transition to noninvasive ventilation for 24 h. The patient was afebrile since Day 2 (maximum temperature 40.5°C, Day 1). Thrombocytopenia was minimal at 20 G/L requiring two platelet transfusions and then gradually improved from Day 2 (Table 1). Hepatic cytolysis and cholestasis gradually decreased and returned to normal values on Day 7, whereas prerenal insufficiency resolved quickly (Table 1). The patient was treated with doxycycline for 14 days, with a complete recovery. Subsequent PCR performed on blood, pleural effusion, and endotracheal secretions showed a progressive decrease of the number of copies of *R. typhi* (Figure 2).

**Table 2:** Microbial tests performed.

Blood	Bacterial culture
	‐ Anaerobic
	‐ Aerobic
	Levure culture
	PCR
	‐ Eubacteria
	*- Leptospira*
	*- Mycoplasma pneumoniae*
	*- Chlamydia pneumoniae*
	*- Legionella pneumoniae*
	*- Staphylococcus aureus*
	*- Rickettsia* ssp.
	*- Typhus group* (*Rickettsia typhi*/*Rickettsia prowazekii*)
	Serology
	‐ Dengue IgG/IgM
	‐ Rickettsia IgG/IgM
	Parasite
	‐ *Plasmodium* screening (immunochromatography, microscopy, and PCR)

Endotracheal suction	Bacterial culture
	*PCR*
	*- Influenza virus A/B*
	*- RSV*
	*- SARS-CoV2*
	*- Pan-enterovirus*
	*- Mycoplasma pneumoniae*
	*- Chlamydia pneumoniae*
	*- Legionella pneumoniae*
	*- Plasmodium* ssp.
	*- Rickettsia* ssp.
	*- Typhus group* (*R. typhi*/*R. prowazekii*)

Urine	Bacterial culture
	*Francisella tularensis* PCR

Pleural effusion	Bacterial culture
	PCR
	‐ Eubacteria
	*- Mycoplasma pneumoniae*
	*- Chlamydia pneumoniae*
	*- Legionella pneumoniae*
	*- Francisella tularensis*
	*- Rickettsia* ssp.
	*- Typhus group* (*R. typhi*/*R. prowazekii*)

## 3. Discussion

Upon the admission in the PICU, diagnosis of septic shock was clear but not its etiology. Given the history of exposure to pathogens present in sub‐Saharan Africa, the diagnostic reasoning was guided by the local epidemiology. Possible diagnoses included malaria, dengue fever, hemorrhagic fever, leptospirosis, rickettsial infections, typhus, and enteric fever, paying particular attention to rapidly diagnose malaria because of its high mortality risk [5]. Malaria was quickly ruled out by rapid antigen test, blood smear, and PCR. Doxycycline was added to treat possible rickettsiosis and leptospirosis because they were compatible with the symptoms of fever, headache, cough with hemoptysis, hepatosplenomegaly, and thrombopenia in a traveler from Kenya. Two days later, PCR confirmed a rickettsial infection due to a bacterium of the typhus group. Exposure history, clinical presentation, and serology suggested that the disease was murine typhus due to *R. typhi* (rather than epidemic typhus due to *R. prowazekii*).

In Kenya and Somalia, murine typhus is endemic, whereas epidemic typhus is mainly documented in subjects experiencing very poor sanitation conditions (refugee camps and some jails). There are no data on the prevalence of murine typhus in Somalia or Kenya. Although the disease is endemic in sub‐Saharan Africa up to the Horn of Africa and its vectors and reservoirs (rodents and their fleas) are widespread in these countries, including in large towns, the lack of medical infrastructure means that many cases of fever remain of unknown etiology and that murine typhus is likely significantly underdiagnosed in Somalia and Kenya.

In Switzerland, typhus is very rare, with only six documented cases at the Lausanne University Hospital over a 20‐year period (unpublished data). The few diagnosed cases of murine typhus are generally imported infections in individuals returning from tropical regions (primarily Africa and Asia). Considering that our university center diagnoses between 33% and 66% of murine typhus cases observed in Switzerland via PCR or serology, the incidence of (imported) cases diagnosed in Switzerland is approximately 0.45–0.9 cases per year. However, incidence is rising worldwide due to global warming, increased human contact with vectors, more tropical travelers, and immigration [3]. At the European level, seroprevalence in the general population is also very low outside of Mediterranean endemic foci and, even close to zero, if we do not consider individuals exposed during travel to endemic areas [2].

The diagnosis of typhus can sometimes be challenging (especially in the absence of rash), given its rather nonspecific symptoms. Murine typhus typically presents with a triad of fever, headache, and rash. However, this triad is observed in only 42% of children according to a systematic review [1]. Other common signs include malaise, chills, anorexia, cough, dyspnea, myalgia, and arthralgia. In children, gastrointestinal symptoms such as abdominal pain, diarrhea, and nausea or vomiting are more frequently reported than in adults, affecting about 25% of patients. Our patient exhibited all these symptoms, but the rash was not obvious due to the dark skin color. In the previously cited study, a rash was detected in only 47.5% patients and was less common in populations with dark complexion.

In most cases, the disease is mild and self‐limiting. However, more severe cases exist. The study by Tsioutis et al. [1] identified that complicated illness, defined by organ failure or the need for hospitalization, occurs in 26% of adults and 15% of children. Among adults, complications include respiratory, neurological, and septic shock (only 0.2%). Complications in children mainly involve the pulmonary or central nervous system. The ICU admission rate is 6% for adults and 1.5% for children. Mortality is less than 1% in adults, with no reported deaths in children in this study. We did not find any evidence of deaths among children in the other studies consulted. In the absence of specific treatment, the fatality rate rises to 4% [6].

A 5‐year prospective case‐series study involving 90 adults indicated that 23 patients (25%) experienced complications, totaling 34 complications, with 15 being pulmonary (13 interstitial infiltrations and 2 pleural effusions), primarily in elderly individuals [7]. This common occurrence of lung lesions is likely due to the tropism of the rickettsia for endothelial cells and typhus should be considered in immigrants or travelers coming from endemic areas and presenting with a severe pneumonia.

Host factors associated with more severe rickettsial infections include age greater than 40 years, male gender, and glucose‐6‐phosphate dehydrogenase (G‐6‐PD) deficiency [6]. Delayed diagnosis is also linked to severe cases [6]. In our case, our 16‐year‐old patient initially presented with rather pediatric symptoms (digestive symptoms). However, the complications that occur are related to adulthood. This can complicate the initial diagnosis and cause a delay in care. Despite his age, he has two risk factors for severe disease: He is male and experienced a delay in diagnosis (initiation of doxycycline on the seventh day of symptoms). He did not have a G6PD deficiency.

Our patient rapidly progressed to septic shock with ARDS and multiple organ failure. In the literature, several cases of septic shock and/or ARDS have been documented, but these remain rare. In a retrospective study over 3 years in Texas, among 90 documented cases, four adults presented considered in septic shock, and no children were reported [8]. Bernabeu‐Wittel et al. [9] described two cases of septic shock and multiple organ failure in adults: one involved pneumonia and septic shock, whereas the other presented with septic shock and ARDS complicated by pulmonary and abdominal hemorrhaging due to disseminated intravascular coagulation. A few other case reports describe septic shock or multiorgan failure, always in adults [10–15].

In our case, respiratory symptoms appeared 5 days after the onset of symptoms, with hemoptysis occurring on the seventh day of symptoms. Then, he evolved in ARDS and septic shock. The CT scan performed at the admission shows a fluffy appearance consistent with alveolar hemorrhage. In Tsioutis et al.′s [1] systematic review, cough was observed in 26% of adults and 15% of children; pneumonitis in 7% and respiratory failure in < 0.3%. Another review reports that 30% of cases present respiratory symptoms, but respiratory distress or failure was observed in only seven patients out of 621, two of whom developed ARDS with a fatal outcome. The prevalence of radiological abnormalities was 16.7%, with the majority consisting of either bilateral or unilateral interstitial infiltrates [16].

The pulmonary alterations in murine typhus may be associated with compromised pulmonary microcirculation, explained by the tropism of rickettsia for endothelial cells. Autopsies of two cases of death due to rickettsiosis are very enlightening. Walker described the autopsy of an elderly person who died from septic shock related to *Rickettsia*. The necropsy revealed widespread damage to the blood vessels, with the most notable lesions being diffuse alveolar damage associated with pulmonary edema, interstitial pneumonia, hyaline membranes, and alveolar hemorrhages. Other organs also showed signs of vasculitis and perivasculitis. Immunofluorescent *R. typhi* was demonstrated in the endothelial cells of the lungs, brain, kidneys, liver, and heart, highlighting that *R. typhi* is an obligate intracellular bacillus [17]. Stephens et al. [18] also describes hemorrhagic pneumonia and multifocal small vessel thrombosis in the lungs, skin, and kidneys in the autopsy of a patient who died from multiorgan failure due to rickettsiosis.

Most clinical features of rickettsial diseases are attributed to the disseminated infection of the endothelium, where these bacteria proliferate and induce oxidative stress, resulting in damage to endothelial cells. The most prominent effects of rickettsial infection on endothelial cells include increased vascular permeability, generalized vascular inflammation, edema, enhanced leukocyte‐endothelium interactions, and the release of potent vasoactive mediators that promote coagulation and pro‐inflammatory cytokines [17]. These endothelial lesions in the pulmonary microvasculature can lead, in some cases, to interstitial pneumonia, diffuse alveolar damage, and increased vascular permeability in the lungs, ultimately resulting in alveolar hemorrhage and ARDS, as in our patient. Thrombocytopenia, present in 42% of cases in Tsioutis et al.′s [1] systematic review, also reflects the consumption of platelets in microthrombi.

On the other hand, the cytokine cascade and increased vascular permeability are responsible for the vasoplegia and capillary leak characteristic of septic shock. Early initiation of empiric broad‐spectrum antibiotics is an evidence‐based intervention for reversing the cascade of sepsis and multiorgan dysfunction in critically ill patients [19]. In our case, doxycycline was started immediately upon admission to the PICU, and the clinical evolution was quickly favorable. Of note, doxycycline is the recommended treatment for rickettsial infections in children of all ages. Although tetracyclines have been associated with dental staining in children under 8 years [20], this risk is negligible with short treatment courses, especially when comparing that risk with the severity of the disease. The American Academy of Pediatrics therefore supports the use of doxycycline for short duration [21]. We emphasize the importance of maintaining a high index of suspicion and initiating targeted anti‐rickettsial therapy as soon as *Rickettsia* is suspected, regardless of age.

The importance of early recognition and treatment of murine typhus cannot be overstated, particularly given the limited availability of rapid diagnostic tests. Serological tests remain widely used but only provide a retrospective diagnosis. Moreover, species identification is limited by cross‐reaction. PCR is increasingly used to obtain more immediate results [22]. In our hospital, we have access to PCR analyses, which allow us to make quickly through PCR analysis of various samples (blood, pleural fluid, and endotracheal aspirate), even before receiving the serology results. Only the second serology carried out a week later was able to confirm the diagnosis. The observed decrease in copy number across successive PCR samples provided evidence of a biological response to the treatment (Figure 2) and helped define the treatment duration. Given the poor outcomes of untreated infections, empirical treatment should be introduced when the diagnosis is suspected without waiting serology or PCR results.

## 4. Conclusions

This case highlights the critical importance of promptly suspecting murine typhus and initiating treatment with doxycycline in febrile patients returning from endemic regions to reduce the risk of severe complications and mortality. Awareness of this disease is essential, especially in a world where travel and migration are increasingly common.

Alveolar hemorrhage, rarely reported in literature, is of particular interest and provides a good illustration of the pathophysiology of intracellular replication of rickettsia in endothelial cells. Additionally, the use of PCR proved invaluable for early confirmation of the diagnosis.

## Author Contributions

Pierre H. François and Thalia Leclerc have contributed equally to this work and share the first authorship.

## Funding

Open access publishing was facilitated by Universite de Lausanne, as part of the Wiley–Universite de Lausanne agreement via the Consortium of Swiss Academic Libraries.

## Disclosure

The authors reviewed and edited the content as needed and took full responsibility for the content of the publication. All authors have read and approved the final version of the manuscript. The corresponding author had full access to all of the data in this study and takes complete responsibility for the integrity of the data and the accuracy of the data analysis. The lead author affirms that this manuscript is an honest, accurate, and transparent account of the case being reported and that no important aspects of the case have been omitted.

## Consent

Written informed consent was obtained from the patient for publication of this case and any accompanying images or clinical information.

## Conflicts of Interest

The authors declare no conflicts of interest.

## Supporting information


**Supporting Information** Additional supporting information can be found online in the Supporting Information section. The CARE‐checklist has been completed and is available in the “Supporting Information” section, in accordance with the submission requirements.

## Data Availability

The data that support the findings of this study are available on request from the corresponding author. The data are not publicly available due to privacy or ethical restrictions.
