# Supplementary material for: A 16‐Year‐Old Boy With ARDS and Septic Shock, an Atypical Presentation of Murine Typhus: Case Report and Review of the Literature
Source: Case Rep Crit Care. 2026 Sep 10;2026:1267884. doi: 10.1155/crcc/1267884 (PMC13560501; doi:10.1155/crcc/1267884)
Supplement: Supplementary file 1 [file CRCC-2026-1267884-s001.pdf]

CARE case report guidelines

CARE Checklist of information to include when writing a case report

(cc) BY-NC-ND

Topic	Item	Checklist item description	Reported on Line
Title	1	The diagnosis or intervention of primary focus followed by the words "case report"	1-3
Key Words	2	2 to 5 key words that identify diagnoses or interventions in this case report, including "case report"	18
Abstract (no references)	3a	Introduction: What is unique about this case and what does it add to the scientific literature?	21-28
	3b	Main symptoms and/or important clinical findings	25-27
	3c	The main diagnoses, therapeutic interventions, and outcomes	27-28
	3d	Conclusion—What is the main "take-away" lesson(s) from this case?	29-31
Introduction	4	One or two paragraphs summarizing why this case is unique (**may include references**)	34-48
Patient Information	5a	De-identified patient specific information.	50-54
	5b	Primary concerns and symptoms of the patient.	51-52 ; 55-57 ; 61-64
	5c	Medical, family, and psycho-social history including relevant genetic information	52-54
	5d	Relevant past interventions with outcomes	53 (none)
Clinical Findings	6	Describe significant physical examination (PE) and important clinical findings.	58 ; 65-68 ; 89-90
Timeline	7	Historical and current information from this episode of care organized as a timeline	50-116 and Figure 2
Diagnostic Assessment	8a	Diagnostic testing (such as PE, laboratory testing, imaging, surveys).	Tables 1 and 2, Figure 1, 58-59; 60-75; 81-84; 99-107
	8b	Diagnostic challenges (such as access to testing, financial, or cultural)	99-107
	8c	Diagnosis (including other diagnoses considered)	90-96 ; 119-129
	8d	Prognosis (such as staging in oncology) where applicable	not applicable
Therapeutic Intervention	9a	Types of therapeutic intervention (such as pharmacologic, surgical, preventive, self-care)	65-66; 76-77; 80-85;
	9b	Administration of therapeutic intervention (such as dosage, strength, duration)	87-88; -; 109-110; 114
	9c	Changes in therapeutic intervention (with rationale)	90-96
Follow-up and Outcomes	10a	Clinician and patient-assessed outcomes (if available)	108-116
	10b	Important follow-up diagnostic and other test results	108-116
	10c	Intervention adherence and tolerability (How was this assessed?)	not applicable
	10d	Adverse and unanticipated events	not applicable
Discussion	11a	A scientific discussion of the strengths AND limitations associated with this case report	119-227
	11b	Discussion of the relevant medical literature **with references**.	119-227
	11c	The scientific rationale for any conclusions (including assessment of possible causes)	119-227
	11d	The primary "take-away" lessons of this case report (without references) in a one paragraph conclusion	228-235
Patient Perspective	12	The patient should share their perspective in one to two paragraphs on the treatment(s) they received	
Informed Consent	13	Did the patient give informed consent? Please provide if requested	Yes ☒ No ☐
